# A qualitative study of first time mothers’ experiences of postnatal social support from health professionals in England

**DOI:** 10.1016/j.wombi.2020.10.012

**Published:** 2021-09

**Authors:** Jenny McLeish, Merryl Harvey, Maggie Redshaw, Fiona Alderdice

**Affiliations:** a NIHR Policy Research Unit in Maternal and Neonatal Health and Care, National Perinatal Epidemiology Unit, University of Oxford, Old Road Campus, Headington, Oxford OX3 7LF, UK; b School of Nursing and Midwifery, City South Campus, Birmingham City University, Westbourne Road, Birmingham B15 3TN, UK

**Keywords:** Postnatal care, Social support, Psychological stress, Postpartum period, Midwifery, Nurse-patient relations

## Abstract

**Problem:**

Many women experience the transition to motherhood as stressful and find it challenging to cope, contributing to poor emotional wellbeing.

**Background:**

Postnatal social support from health professionals can support new mothers in coping with this transition, but their social support role during the postnatal period is poorly defined.

**Aim:**

To explore how first time mothers in England experienced social support from health professionals involved in their postnatal care.

**Methods:**

A qualitative descriptive study, theoretically informed by phenomenological social psychology, based on semi-structured, in-depth interviews with 32 mothers from diverse backgrounds. These were analysed using inductive thematic analysis, with themes subsequently mapped on to the four dimensional model of social support (emotional, appraisal, informational, practical).

**Findings:**

There were nine themes connected to social support, with the strongest mapping to appraisal and informational support: for appraisal support, ‘Praise and validation’, ‘Criticism and undermining’, and ‘Made to feel powerless’; for informational support, ‘Is this normal?’, ‘Need for proactive information’, and ‘Confusion about postnatal care’; for emotional support, ‘Treated as an individual and heard’ and ‘Impersonal care and being ignored’; for practical support, ‘Enabling partners to provide practical support’.

**Conclusions:**

Health professionals can play an important role postnatally in helping first time mothers to cope, develop confidence and to thrive, by taking every opportunity to give appropriate and personalised appraisal, informational and emotional social support alongside clinical care. Training and professional leadership may help to ensure that all health professionals are able and expected to offer the positive social support already offered by some.

**Statement of significance**

**Issue**

Becoming a mother for the first time can be stressful and some mothers struggle to cope.

**What is already known**

Social support from health professionals can help new mothers to cope with this transition, but their social support role is unclear.

**What this paper adds**

Appraisal and informational support from health professionals were very important for confidence and coping in first time mothers from varied socio-demographic backgrounds, whether or not they also had social support from a partner, family or friends. Emotional support was valued but had a more limited role. There was minimal practical support from health professionals.

;1;

## 1 Introduction

National guidance in England conceptualises the role of postnatal care to be primarily about support for the transition to parenthood [1]. Some women experience becoming a mother for the first time as a time of stress and poor emotional wellbeing, leading to psychological distress (including depression and anxiety) if they feel unable to cope effectively [[2], [3], [4]]. Social support – a person’s perception of the availability of others to provide emotional, psychological and material resources [5] – is an important factor in enabling a successful transition to motherhood [3]. Empirical research demonstrates that effective social support from health professionals can assist new mothers in coping with the stress of new parenthood by increasing their parenting confidence [[6], [7], [8]].

Social support is a multi-dimensional concept, commonly analysed as having four functional aspects — emotional, appraisal (affirmational), informational and practical [9]. Emotional support consists of words or actions that show love, liking, empathy, respect and trust, leading the recipient to believe that they are cared for, esteemed and valued [9]. Appraisal or affirmational support is the communication of information to enable positive self-evaluation, specifically affirmation of the rightness of what the recipient has done or said [9], and thus a key ingredient of constructive feedback [10]. Informational support is information provided to another at a time of stress [9], including information about a baby’s health and development [10]. Practical or instrumental support is the provision of tangible goods, services or aid [9], in this context specifically help with caring for the baby [10].

There are contrasting findings from different countries about the principal aspect of social support mothers report receiving from health professionals postnatally, for example practical or informational support on postnatal wards in Finland [11], and informational support in Ireland [8] and in the community in Finland [6]. This is complicated by different definitions, for example Salonen et al. [11] categorise ‘infant-care instructions’ as part of appraisal support and ‘directions for infant feeding’ as part of practical support, while Tarkka et al. [6] categorise information and advice on child development as part of affirmation support.

New mothers may receive social support from a variety of sources apart from health professionals, including their partner, parents, other family members, friends, neighbours, and community volunteers [[6], [7], [8],^12^]. They may want and receive different aspects of support from informal and formal sources, so one does not replace the other [7,8]. Where the aspect of support received does not match the aspect of support desired, it may be ineffective or may increase rather than diminish stress [5]. In particular, where health professionals do not provide the emotional and affirmational support that new mothers want in the immediate postnatal period, interactions with health professionals may themselves become an additional source of stress instead of buffering the stress of new motherhood [7].

Mothers who give birth in England usually have access to free National Health Service postnatal care. This includes support from midwives and maternity support workers on a hospital postnatal ward or birth centre and in the community, a health visitor who takes over from the midwifery team as the lead practitioner approximately 10–14 days after birth, and a general practitioner who assesses the baby and mother at 6–8 weeks [1]. The social support role expected of health professionals in the postnatal period is poorly defined, but every interaction with a health professional in the postnatal period has a potential social support meaning for the mother, and being aware of these meanings these will enable health professionals to avoid harm and maximise their positive impact on maternal wellbeing [10]. In order to deepen understanding of their social support role and how it can contribute to maternal wellbeing in the transition to motherhood, this article explores how first time mothers in England experienced different aspects of social support from health professionals involved in their immediate postnatal care in the hospital or birth centre and in the community. It reports research that is part of a programme of work on first time mothers’ expectations and experiences of postnatal care that includes an online survey, antenatal interviews and a qualitative longitudinal study, which have been reported separately [[13], [14], [15]].

## 2 Participants, ethics and methods

### 2.1 Study design

This was a qualitative descriptive study [16], based on semi-structured, in-depth interviews, theoretically informed by phenomenological social psychology which focuses on participants’ lived experiences and subjective meanings of social interactions [17]. This ‘low-inference’ [16] design was chosen because the purpose was to explore participants’ own perceptions and thus to stay close to their accounts [17], while acknowledging the role of both participants’ understandings and the researchers’ interpretations in the production of knowledge [18]. Throughout the research process, the researchers worked with a reflexive awareness of their own perspectives on the transition to motherhood and postnatal care, based on professional knowledge and diverse personal experiences.

The University of Oxford Medical Sciences Inter-Divisional Research Ethics Committee (reference R52703/RE001) approved the study.

### 2.2 Participants

The interviews reported in this paper were second (postnatal) interviews within a qualitative longitudinal study. Participants were women who had given birth to a live baby or babies in England in the past four months, and had previously taken part in a first (pregnancy) interview. The original recruitment criteria for the pregnancy interviews were: currently in the third trimester of pregnancy; aged 16 or over; planning to give birth in England; and had not given birth previously. Purposive maximum variation sampling [19] was used to recruit women with a range of socio-demographic characteristics, with a particular emphasis on seeking diversity in age, ethnicity, and socio- economic status using postcode quintiles [20]. Multiple recruitment strategies were used to include women who are less likely to participate in research [21] and in particular younger women and women living in more deprived areas, who are less likely to respond to maternity surveys [22]. These were: (1) an invitation at the end of an online survey about expectations of postnatal care, promoted on social media by parenting organisations; (2) an in-person invitation from a researcher to women attending three sessions of a young mothers’ antenatal group and two sessions of a free antenatal exercise class, each run by a community group in a different area of high deprivation; (3) an advertisement circulated on social media by a multiple birth charity. There was intentional over-recruitment at the stage of pregnancy interviews, to allow for the likelihood that some participants would drop out before the postnatal interviews and to ensure demographic variation. The only prior relationship between the researchers and the participants was the research relationship established during the pregnancy interviews.

Thirty two women took part in the postnatal interviews reported here, when their babies were 7–15 weeks old (median 11 weeks). A further eight women who had taken part in pregnancy interviews could not be contacted after birth. Background information about participants in postnatal interviews is shown in Table 1.

**Table 1:** Background informationabout participants.

	**Number of participants n = 32 (% rounded)**
**Age**	
Under 25 years	3 (9%)
25–29 years	10 (31%)
30–34 years	10 (31%)
35+ years	9 (28%)
**Ethnicity**	
White	26 (81%)
Mixed or multiple ethnicity	4 (13%)
Black	1 (3%)
Asian	1 (3%)
**Country of origin**	
UK	27 (84%)
Outside UK	5 (16%)
**Index ofmultiple deprivation quintile postcode classification** [23]	
Quintile 1 (most deprived)	5 (16%)
Quintile 2	4 (13%)
Quintile 3	10 (31%)
Quintile 4	6 (19%)
Quintile 5 (least deprived)	7 (22%)
**Relationship status**	
With husband/partner	30 (94%)
Single	2 (6%)
**Physical or mental health condition**	
No	25 (78%)
Yes	7 (22%)
**Urban/rural location**	
City/large town	25 (78%)
Village/countryside	7 (22%)
**Place of birth**	
Birth centre	4 (13%)
Labour ward	27 (84%)
Home	1 (3%)
**Type of birth**	
Spontaneous vaginal birth	13 (41%)
Unassisted vaginal birth following induction	2 (6%)
Assisted birth	9 (28%)
Caesarean section	8 (25%)
**Length of stay on postnatal ward or at birth centre (n = 31)**	
<1 night	1 (3%)
1 night	11 (35%)
2 nights	11 (35%)
3–4 nights	3 (10%)
5+ nights	5 (16%)
**Partner/family member allowed to stay overnight on postnatal ward or at birth centre (n = 30)**	
Yes	18 (60%)
No	12 (40%)

### 2.3 Data collection

Data were collected through semi-structured qualitative interviews between January and July 2018. Topics included the help received from health professionals and others postnatally in hospital or birth centre and in the community, whether this matched what the mother felt she needed, and the impact the mother felt the postnatal care had on her. At the end of the first (pregnancy) interview, participants were asked for permission for the researcher to contact them approximately six weeks after their baby’s due date to arrange a second (postnatal) interview, either face-to-face at a time and place of their choice, or by telephone. Twenty nine postnatal interviews were by telephone and three were face-to-face, ranging in length from 21 to 56 min (mean 37.5 min). Informed consent to participate had been obtained before the pregnancy interview through a signed consent form if face-to-face, or given orally and recorded in writing when interviews were carried out by telephone; participants were reminded of this at the start of the postnatal interview and asked to confirm verbally whether they continued to consent. Participants were offered a shopping voucher worth £15 at the end of the interview, to thank them for their time. All interviews were carried out in English, although interpreting support was available if required. No one else (apart from babies) was present at the face-to-face interviews. Interviews were audio-recorded and fully professionally transcribed, with each participant being given an anonymous identifier beginning with PNC for ‘postnatal care’.

Data collection continued past the point where saturation was reached in these postnatal interviews (that is, participants were repeating what had been expressed by previous participants and there were no new codes or themes). Instead, all women who took part in pregnancy interviews were invited to take part in postnatal interviews. This was done to preserve the demographic variation of the initial sample, and to honour the commitment made to participants in the original participant information that they would be contributing to a qualitative longitudinal study through interviews before and after birth.

### 2.4 Data analysis

Inductive thematic analysis was carried out in parallel with ongoing data collection. Interview transcripts (which were the units of analysis) were checked against the audio-recordings, and read and reread for familiarity. Data were coded using NVIVO software. Codes were refined and combined as data collection continued, and themes describing manifest content were developed, using constant comparison to reconsider early analysis in the light of subsequent interviews. In order to explore the specific functional aspects of social support in postnatal interactions, themes were then mapped deductively onto the four dimensional model of social support [9], as widely used in social support research including Leahy-Warren’s concept analysis of social support for new mothers [10]. To increase the validity of the analysis, one researcher analysed all the transcripts and another analysed a subset; codes and themes were discussed and agreed. De-identified excerpts of interviews were selected to illustrate the findings.

## 3 Findings

**Fig. 1:**
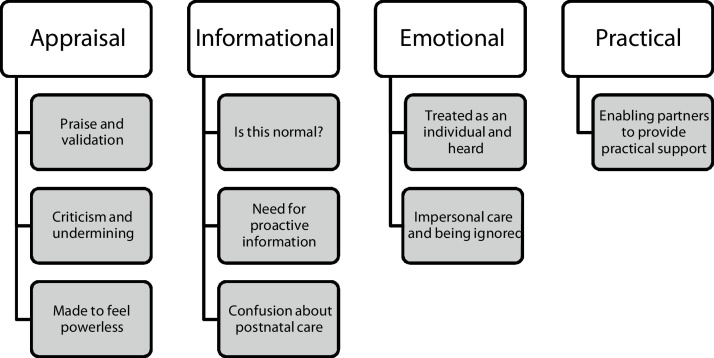
Themes and functional aspects of support interactions from professionals.

There were 9 themes identified relevant to social support. Fig. 1 shows how these themes mapped on to the four functional aspects of social support. The strongest mapping was to appraisal and informational support. There were no differences identified according to socio-demographic background of mothers.

### 3.1 Appraisal support

There were three themes related to appraisal support: ‘Praise and reassurance’, ‘Criticism and undermining’, and ‘Made to feel powerless’.

#### 3.1.1 Praise and validation

Many mothers described how, as first time mothers, they had no way to evaluate themselves: *“I didn’t quite know what I was doing and what was right and what wasn’t right”* (PNC110). They highlighted the importance of being reassured by midwives or health visitors that they were doing things ‘correctly’: *“Praise, but not in a patronising way … ‘You’re talking to him, you’re engaging with him, he’s growing really well.’ … She just made me feel really good about what I was doing”* (PNC060). Positive appraisal was felt to be much more meaningful when it came from a professional who was seen as objective, compared to friends and family who might be positive out of kindness: *“And it’s different, when one professional says it, than when it’s like your mum or your friend says [insincere tone], ‘Oh you’re doing great!’”* (PNC055). They were worried about being seen as “*a really over-exaggerating mother”* (PNC105), so it was affirming to hear from professionals that having lots of questions was completely normal: *“I expected for myself that I should know how to do all of this, and that was helpful at that stage to be told, ‘Nobody expects you to know what you’re doing, you haven’t done this before’”* (PNC603). One mother commented that receiving only basic postnatal support had in itself felt like an affirmation of her parenting competence: *“The fact that they haven’t felt that I’ve needed any extra help has given me confidence that I’m doing it right”* (PNC224).

There were differences in the extent to which mothers believed that there was a ‘right’ way to carry out parenting tasks which they could master, and that health professionals were the experts in what this was. For example, one mother had asked midwives to watch her breastfeeding: *“Just correct anything I’m doing wrong or [the baby] ’s doing wrong”* (PNC055). Some others strongly preferred to be given non- directive advice and to make their own choices. Health professionals’ praise was often linked to how well the baby was growing and many mothers also accepted this as a reflection of their own performance as parents:

*“Measurements and weight and things like that have all been spot on and I’ve always had nice compliments from midwives and the health visitor …You did get a lot of, ‘Well done, mum,’ pat on the back kind of thing. It was quite a confidence boost”* (PNC158).

This carried the risk that if babies did not feed and gain weight as expected, mothers could feel that they were personally failing: *“I was very tearful and upset about it, and I think that was because I had such strong ideas about what it was going to be like and the experience wasn’t matching up with that*” (PNC603).

#### 3.1.2 Criticism and undermining

Many mothers reported encounters with professionals that disaffirmed their competence and undermined their confidence. They were given negative feedback in ways that left them feeling *“like a little kid that had been told off”* (PNC131), either through direct comments or body language such as eye rolling: *“There’s quite a lot of pressure put on you in the hospital to be this perfect mum… They don’t quite say you’re being a terrible mother, but that’s how it felt … It makes you doubt everything that you’re doing a lot of the time*” (PNC250). This was particularly demoralising to mothers who were criticised when they were following a different health professional’s advice:

*“She said, ‘What are you doing? You shouldn’t be doing that.’ We’d been told two days ago that that’s what we should be doing, it was then really confusing to be told something else, and she was quite abrupt in her manner and it was a bit of a knock to the confidence … It doesn’t make you feel very good”* (PNC129).

Far from being reassured that their questions were welcomed and normal, some mothers described being made to foolish or incapable when they tried to get answers: “*It felt like I was being put back in my place with the sort of questions I was asking my health visitor”* (PNC158). One mother had felt criticised and reprimanded in several encounters with professionals, and she reflected on the irony that they asked questions about maternal mental health but were lacking in self-awareness about the negative impact of their ordinary interactions with mothers:

*“Why was she so angry at me about asking? … They concentrate so much on looking for signs of postnatal depression but don’t understand how they can just add to that, because then you’re left feeling like an idiot for asking a simple question”* (PNC131).

Where a mother was struggling, it was very upsetting to feel she was being judged by someone who did not know the individual facts but was just giving generic advice, as in this example of a mother who was told by a health visitor at the six week check that combination feeding was not the best choice:

*“She didn’t know the back story, she didn’t know us … On day 5 when we had the midwife it was like 90% formula, hardly any boob at all, and now it was about 75% breastfeeding and 25% formula, and it was something that we’d worked really hard at and it had taken a long time, and at a point I was really proud of, and then I just met her and she very quickly made it feel like it just wasn’t good enough.”* (PNC194).

Another mother’s experience illustrates the sensitivity of new mothers to the possibility of implied criticism, when the health professional’s remark was intended to be affirming but sounded insincere because it was not related to the mother’s actual needs. This mother had just had an assisted birth:

*“[The midwife] said, ‘Well, don’t feel disappointed with yourself’ … She was saying it more as like a platitude than a genuine, ‘You’ve done amazing.’ But I think because I wasn’t feeling disappointed or feeling like things had gone badly, her saying that then made me think, ‘Oh, maybe she’s thinking it didn’t go that well… actually maybe I should be disappointed’…I’m still upset about it”* (PNC012).

#### 3.1.3 Made to feel powerless

A few mothers reported that some health professionals had acted as though they had power over the mother’s and baby’s bodies that the mother had no right to challenge. Although this did not involve *explicit* criticism of the mothers (as in the previous subtheme), they had understood an implication that the mother was not seen as competent to make her own choices: *“They think, ‘Oh yes, we’re God and you just trust us … We know, you don’t know anything’”* (PNC702). Most of these situations related to professionals either administering medication or carrying out procedures without the mother’s informed consent: *“Nobody told me that [my baby] had a chest X-ray or asked if that was okay”* (PNC046). Others related to mothers being required to stay in hospital for extended periods without a clear explanation of why this was necessary, and feeling scared to disobey: *“We weren’t allowed to leave the ward … I was getting to the point where I was just going to be like, ‘I’m just going to discharge myself,’ then I thought they might think I’m an unfit mother”* (PNC250).

One mother had experienced multiple forms of disempowerment while in hospital, which had a dramatic impact on her emotional wellbeing, and affected her interpretation of routine aspects of postnatal care like electronic tagging:

*“They had an electronic tag on [the baby]’s leg, which at first I thought was so they don’t steal your baby, and then I realised we can’t leave. So, I felt really trapped, like we were in prison … No one explained anything … They came and did a lumbar puncture at some point and didn’t tell me why. I thought [the baby] was going to be paralyzed … And people kept coming with more and more drugs for us... I didn’t want to be on antibiotics, and they didn’t give me an option. I wish I had known that I had that power [to give consent]… I ended up feeling like I’d failed [the baby], and at the beginning of his life story. I was so sad.”* (PNC702).

### 3.2 Informational support

There were three themes related to informational support: ‘Is this normal? ’, ‘Need for proactive information’, and ‘Confusion about postnatal care’.

#### 3.2.1 Is this normal?

Most mothers had checked with a professional about an aspect of their own health or their baby’s health or development that they were worried about, asking the question *“Is this normal…?”* Generally they had received the reassuring information that they needed, which also boosted positive self-appraisal. They strongly valued having ready access to professionals who could answer their questions:

“*I could always ring the postnatal ward in the hospital if there were any problems … Nearly every day that first week we had a midwife come, and that was really helpful and we were able to write down all our questions … I don’t think we would have coped nearly half as well without them.”* (PNC110).

Most had received advice about feeding their babies, and a few commented that they had also received helpful information about practical aspects of baby care from an individual midwife or health visitor in response to specific challenges: *“The best thing [the midwife] did for us was to give us advice that wasn’t ‘midwife advice’, that wasn’t necessarily about the weight of baby, the jaundice. It was more about, ‘Here’s something you can do so that you can get more sleep’”* (PNC091).

Confidence in professional advice was seriously eroded for the many mothers who reported receiving conflicting information from health professionals, concerning issues such as how to breastfeed, expressing milk, swaddling, keeping babies warm, safe sleeping, self-care after a caesarean, oil for the baby’s skin, jaundice, tongue tie and vitamins. This made their advice appear to be anecdotal and unreliable:

*“You would have a different midwife every eight hours and they had very different opinions of the clothes he should have on, how I should be feeding him, what I should be doing … if people are telling you different things, you don’t know who to listen to.”* (PNC701).

A couple of mothers said they did not mind this inconsistency: *“The plus side of that is you get two different opinions*” (PNC105). Diverse opinions opened space for a more confident mother to make her own choice, but she would also check the information against independent sources: *“It seemed they had a favourite personal preference for doing things certain ways … it did mean that I could take what I wanted from whichever midwife… I definitely have continued to look stuff up myself as well”* (PNC224).

Despite multiple experiences with advice that they found questionable, in principle most of the mothers wanted to get their information from health professionals or NHS- approved resources, believing that this was likely to be more reliable, up to date and evidence- based than information from other sources. Nonetheless all the mothers described how they sought out different kinds of postnatal information from different people. Even if they saw health professionals as approachable and accessible, they were selective in what they asked them, because they did not want to bother them with what might turn out to be unimportant questions: *“I’ve relied more on my friends to ask them, because as much as [the health visitor] said you could call her any time, you kind of feel as though you can’t”* (PNC192). Sometimes this was a strategic choice to prioritise professional information on specific topics: “*The health visitor it’s pretty much all about health, and then the other mums it’s about life”* (PNC240). Other reasons for mothers’ reluctance to ask questions are explored in the next subtheme.

Very few mothers had turned to their own mothers as reliable sources of information: *“Quite a lot of the advice has changed from when she had me”* (PNC272). Generally they asked other recent mothers instead, in person, online or using group messaging. Some commented on the risks of relying on peers instead of professionals for information about ‘normality’, when those mothers might be trying to meet their own needs for affirmation either through boasting: *“There’s a lot of competition, ‘My baby’s done this, my baby’s done that’”* (PNC158), or competitive negativity: *“I don’t tell them my baby sleeps so well, because I want to make friends. Everyone [at the baby group] really likes to complain and it’s like one- upmanship on how bad things are: ‘My baby only sleeps for two hours at a time!’ ‘Well my baby only sleeps for half an hour!’”* (PNC105).

#### 3.2.2 Need for proactive information

Although they valued the reassurance and advice they received, mothers identified a need for new mothers to be given important information in advance. Some had been given comprehensive written information: “*I got about* 6000 *leaflets from the health visitor when she first came, on every possible thing” (*PNC501). They questioned whether this format was useful to an overwhelmed new mother who would never have time to read it all to find what she needed, or was merely intended to tick the box that information had been given. What they wanted instead was proactive and concise information about key postnatal problems and typical scenarios for mother and baby, including crying, sleep and feeding:

“*I think they just try to cover themselves with lots of paper giving. At the time after you’ve given birth, you’re not going to be prepared to read all those leaflets, you’re struggling to keep your eyes open … A classic ‘this is normal’ guide would probably be helpful, and a solution page. So, ‘Is this normal to have this?’ and, ‘This is what you can do’”* (PNC102).

There were several overlapping reasons for wanting health professionals to take responsibility for giving mothers relevant information, rather than waiting for mothers to ask. Some were inhibited from asking questions when they did not know the health professional, because of the risk of encountering someone judgmental: *“It’s quite hard to ask someone questions without feeling bad or stupid in yourself when you’ve literally just met them”* (PNC152). Some pointed out that first time mothers might find it hard to articulate a problem, because everything was unfamiliar: *“I didn’t know what the question was. Because if I had just said that the baby is crying, that wouldn’t have been helpful*” (PNC704). This also applied to making the best use of routine contacts with health professionals: *“She was asking if there was anything that I wanted to ask, and sometimes I find it a little bit difficult to know what I want to know”* (PNC272). Another key reason for wanting to be given information before a problem arose was to prevent the considerable stress of worrying about the situation:

*“We didn’t really sleep that first night because we were just watching to make sure [the baby] didn’t choke herself … the midwife on the triage [helpline] said, ‘Oh it just sounds like the mucus, it’s very normal,’ So that was good, but we were both left thinking, ‘If it’s so normal, why didn’t anyone tell you to expect it?’”* (PNC055).

The biggest category of difficulties that mothers said they had not been warned about were related to feeding, particularly that latching on might be painful in the early days of breastfeeding, and the frequency with which a newborn might feed. Advance information about the reality of breastfeeding – *“a bit more honesty”* (PNC060) – would have prevented the loss of parenting confidence when mothers assumed they were to blame:

*“Nobody had really explained cluster feeding and growth spurts, so I just felt like she wasn’t getting enough milk and I was failing her. If I’d have been better prepared for that, then I don’t think I’d have panicked as much or had crying sessions.”* (PNC113).

Some commented that it would have been useful if antenatal classes gave much more time to postnatal life: *“Talk about what to expect when you actually have a baby”* (PNC152). By contrast a few noted that in the antenatal period it might be difficult to fully process information about postnatal life: *“People did say to me, ‘Breastfeeding’s really hard.’ I wish someone had smacked me in the face and said, ‘BREASTFEEDING’S REALLY HARD!’ because they did tell me that, but I didn’t understand”* (PNC091).

#### 3.2.3 Confusion about postnatal care

Most of the mothers gave birth in hospital, and a repeated problem was the lack of effective orientation when they went to the postnatal ward afterwards: *“You’re still in this wheelchair with your baby you’ve just given birth to and they wheel you into the room and they start to walk out the door. Both me and my husband were like, ‘Where are you going? You can’t just leave us! What do we do now?’”*(PNC055). Lack of information about what to do in this unfamiliar situation had led to additional stress about the risk of unwittingly breaking hospital rules: *“It was silly things, like I needed to go and get breakfast … and I was like, ‘Am I going to get in trouble for leaving [the baby] on his own?’”* (PNC060). It had also caused confusion about how much support they could ask for from staff, leading some not to get the help they needed: *“I didn’t really realise until after I’d left hospital … how much more help I could have asked for”* (PNC046).

In the community, some mothers reported being given clear information about the postnatal care they could expect. Others had not: *“No one’s contacted me. I don’t even know who to contact in the health visitor clinic. I don’t know if there is a health visitor clinic”* (PNC131). They suggested that it would have been helpful to be given a list of standard postnatal care appointments, with their likely timing, location and purpose.

### 3.3 Emotional support

There were two themes related to emotional support: ‘Being treated as an individual and heard’ and ‘Being processed impersonally or not listened to.’

#### 3.3.1 Being treated as an individual and heard

Some mothers had received personalised and responsive postnatal care which made them feel liked, valued and cared about:

*“[Midwives] gave me 100% of their care. It wasn’t just their job that they’d done every day for the last however many years: I was really an individual with my own needs … Like you were the only person that mattered at that point … If I texted [the health visitor] to say that [the baby]’d put on weight she’d respond with a positive feedback … genuinely pleased with the milestones I had met.”* (PNC270).

Where the mother had received care from the same midwife on more than one occasion (which applied to only a few), emotional support could be expressed in the context of a relationship: *“[The midwife] was obviously pleased to see me and pleased to see [the baby] and see how well he was getting on … I remember that being particularly special”* (PNC240). Emotional support could also be experienced during one-off interactions where the health professional demonstrated concern and regard by their demeanour: *“I definitely felt [the midwife] was championing my needs and she genuinely cared”* (PNC184). Mothers who believed that professionals had gone beyond their strict responsibility felt cared for and safe: “*The midwives were supposed to finish at the time that the baby went to hospital, but they made sure they called again just to make sure everything was okay”*(PNC078).

Both in hospital and in the community, mothers felt emotionally supported when health professionals listened to them. Some mothers praised individual health professionals who had conveyed sincere interest in their wellbeing by giving them full attention, despite being very busy:

*“She was rushing a bit, but when she asked how my mood was and I said, 'Actually I have been feeling a bit low,'... she really took time, and actually sitting down on the end of my bed… That made it feel really open and okay to talk.”* (PNC603).

#### 3.3.2 Being processed impersonally or not listened to

Many mothers had received inattentive, functional care, particularly on busy postnatal wards, which undermined feelings of safety:

*“It just felt like you were being handed around like a rugby ball, chucked from player to player …They had so many things to do that something could easily be missed. That put you on tenterhooks for the entirety of your stay.”* (PNC184).

This could also be very disappointing for women who had just gone through the life-changing experience of becoming a mother for the first time: *“The after bit in hospital, I thought it would feel a bit more special… we were just shoved off to one side… I wouldn’t say that they really knew who we were”* (PNC189).

These mothers mostly attributed emotionally unsupportive care to staff workloads: *“They were just so exhausted and stretched that they didn’t get a chance to care as much as they could”* (PNC189). It was nonetheless demoralising to be processed impersonally, for example through a confusing hospital discharge less than 6 h after birth:

“*When I literally was verging on 48 hours without any sleep … that lady then came in, she spoke really fast, and just basically went through this sheet of paper… I didn’t know what was going on … then it was like, ‘If you can pee, you can go home.’ So, I peed and I went home”* (PNC152).

Mothers’ emotional wellbeing was also undermined when health professionals did not listen to them, for example when their concerns about their baby or their body were dismissed: *“I told about 10 different people about my blood pressure, I told about 10 different people about him not feeding but … they weren’t having any of it … I felt awful”* (PNC250). These new mothers needed their feelings to be acknowledged and accepted as they learned to cope with motherhood. A mother who was experiencing mental health difficulties, including flashbacks to a traumatic birth and hallucinations, described how professionals did not pay attention to her subjective experience of exhaustion and worry. Instead their attempts to normalise her experience felt to her like they were minimising its significance:

*“These women who have been working in this industry for 20-plus years and think that you’re being a bit pathetic for being in tears …A lot of my mental health has been linked to my lack of sleep …They’ve just completely dismissed it… It’s almost like, ‘Every woman’s exhausted after they give birth, deal with it’”* (PNC131).

### 3.4 Practical support

There was one theme: ‘Enabling partners to provide practical support’.

#### 3.4.1 Enabling partners to provide practical support

Mothers had received very little direct practical support from health professionals. A couple said that staff had helped to calm their crying baby or bottlefeed a twin, but the great majority had not received any practical support at all. This had been particularly challenging and disappointing for mothers who were recovering from a caesarean: *“On the one hand they’re like, ‘Try and rest,’ but then on the other hand they’re like, ‘You still need to do everything that you need to do for your baby’”* (PNC194). Instead, mothers in this study had relied on their partners, family, friends, neighbours and communities for all their practical postnatal support in hospital and at home, particularly while they were recovering from birth interventions: *“In those first couple of days when I couldn’t get up [my partner] did pretty much everything*” (PNC501).

Many mothers commented on the ways in which health professionals and the rules they operated could obstruct the practical support role of partners and family members on postnatal wards. Where they were not allowed to stay with the mother overnight, the absence of their practical support could motivate the mother to leave hospital sooner than she might want: *“I didn’t want to spend another night in the hospital on my own without my husband … just having the help at night time”* (PNC105). Where partners were allowed to stay with the mother all the time, the postnatal ward was not a hospitable environment for them, as most had to sleep on a chair or on the floor, and were not allowed to eat the food or use the shower. This seemed to mothers to undervalue their role and create a barrier to their ability to provide this essential practical support: “*At the birth centre, the person attending, they also are given are a comfortable place to take rest, because that person has to take care of the new mother, right? At the hospital this was completely missing*. *This was very strange”* (PNC704). As a result some partners felt they had to go home overnight to sleep, even though they were allowed to stay. One mother described how her partner was actively made to feel unwelcome by the way staff enforced the rules:

*“He had been awake with me for over 24 hours … then on the ward the partners weren’t allowed to lay down anywhere. Even if I was up with the baby and happy to be up with the baby, and he was on the bed to have a 10 minute nap or something, they’d come and wake him up and shoo him off again and tell him not to do that.”* (PNC250).

## 4 Discussion

Previous research has identified that the majority of mothers are satisfied with their postnatal care in England, but a substantial minority report problems with hospital postnatal care such as insufficient and inconsistent information, poor quality interactions with staff including derogatory comments, and lack of respect for choices [23,24]. Similar problems have been reported internationally and about postnatal care in the community [[25], [26], [27], [28], [29], [30], [31]]. Walker et al. [32] argue from the results of their qualitative systematic review that the key to supporting a successful transition to motherhood is the ability of women and midwives to connect, and that this can best be achieved through postnatal midwifery home care. This study deepens understanding of the ways in which the social support aspects of postnatal care in any setting can be an opportunity to enhance first time mothers’ confidence and skills, or alternatively can undermine mothers’ ability to cope and thrive in the transition to parenthood. It highlights the value that first time mothers from a range of demographics attach to effective postnatal social support from health professionals, even if they also have social support from family and friends; and emphasises the need for health professionals to use every postnatal contact and conversation as an opportunity to offer care in ways that are not only kind and respectful [1], but are also socially supportive.

The strongest themes in this study related to appraisal and informational support from health professionals, and these are the aspects of social support identified by Leahy-Warren [8] as having the greatest salience for maternal confidence. Building up mothers’ parenting confidence through timely information and positive feedback assists them to cope with the stress of having a new baby, by both helping them to deal with stressors effectively and reassuring them that they can rise to this challenge successfully [5]. The varied experiences of mothers in this study, with some receiving significant social support within postnatal interactions and others very little, reflect the uneven provision of postnatal care within overstretched services in England [33].

Almost all mothers, from a range of socio-demographic backgrounds, described the beneficial impact on their parenting confidence of having their actions affirmed and validated by health professionals, even if they also had this affirmation from other sources. They also described the negative impact of feeling judged, criticised, or actively disempowered, and some were acutely sensitive to perceived as well as actual criticism, including where an attempt at affirmation was not related to the mother’s own needs. The importance of affirmation in the context of breastfeeding has previously been highlighted by Schmied et al. [26]. However, the fact that health professionals often linked their praise to successful breastfeeding or a baby growing well could undermine the confidence of mothers where this was not successful, reinforcing their feelings of grief and failure [34,35]. The powerful subjective significance to mothers of interactions being affirming or disaffirming echoes the findings of Razurel et al. [7], who found that unhelpful interactions with caregivers were the single greatest source of stress in the immediate postnatal period, and Aston et al. [36] and Wilkins [37], who described the sensitivity needed to overcome power differentials between ‘expert’ health professionals and new mothers afraid of being judged by experts as not ‘doing it right’.

New mothers are frequently reported to be overwhelmed by the sheer amount of advice available to them from online and offline sources, and to find it challenging to navigate its reliability [[38], [39], [40]]. The fact that very few mothers in this study relied on their own mothers for postnatal information represents a significant social shift from the patterns identified by Leahy-Warren [8]. They felt they had to ration the questions that they asked professionals and turned primarily to other mothers to fill the information gaps, while recognising that informal sources of information might be unreliable or embody unhelpful ideas about ‘normality’ [38]. Nevertheless, despite the perennial problem of inconsistent professional advice [23], mothers in this study generally wanted to get their information from health professionals or resources recommended by health professionals.

In theory, all new mothers in England should have access to information and advice about self-care and baby care [1], and many mothers described positive experiences of seeking answers to their repeated question *“Is this normal? ”* However they also questioned the way in which they had been either given so much written information that they could not find what they needed, or given very little and invited to ask for more. Like the mothers interviewed by Wilkins [37], they suggested that new mothers should proactively be given concise important information about common problems, solutions and reassurances, and clear information about postnatal care itself. This would alleviate unnecessary anxiety, support mothers who felt too vulnerable to expose their lack of knowledge to potentially judgemental health professionals, and protect mothers from the fear of being reprimanded for failing to navigate the unfamiliar system correctly. Although the need for more realistic antenatal preparation for the practical realities of parenthood has been identified many times [41], this research suggests that effective antenatal preparation should sit alongside information available postnatally in a form that new mothers find useful.

Schmied et al. [26], in their metasynthesis of breastfeeding support, distinguish between a ‘facilitative style’ where professionals give realistic, accurate, and proactive information that can both pre-empt and respond to worries, and a ‘reductionist approach’ where professionals give standard information didactically and disregarding the mother’s own views or needs. Other researchers have emphasised how the way in which information is given to new mothers is as important as its content [38,42]. This study reinforces the importance of a facilitative and individualised style across all aspects of postnatal information, even where the mother herself believes that there is a ‘right’ way to look after her baby and that she needs guidance from experts on how to do this.

The current policy aim in England is for most women to have continuity of midwifery carer through the antenatal, intrapartum and postnatal periods, but fewer than one in ten women currently have postnatal care from at least one midwife who was involved in both their antenatal and intrapartum care [24]. Mothers in this study described how emotional support could occur in the context of an ongoing relationship in the few cases where that existed, but it could also occur when one-off interactions were managed skilfully by a health professional. Kind, respectful, empathetic interactions had made them feel safe, valued and cared for, while superficial, rushed interactions had made them feel insignificant and demoralised at a life-changing time. For mothers who were experiencing difficulties, there could be a fine line between having their problems normalised by professionals and feeling that they had been minimised, as also reported by Razurel et al. [7]. This underscores the importance of listening carefully to mothers’ concerns and responding sensitively.

Although three quarters of mothers surveyed by the Care Quality Commission in 2019 reported that their partner or another companion was able to stay with them all the time [24], this study illustrates how the policy intention could be undermined if there were no facilities to accommodate partners and companions, or if they were made to feel unwelcome, leading some mothers to leave the postnatal ward sooner than they wanted in order to have the practical support which was not available from professionals.

### 4.1 Strengths and limitations

It was a strength of this research that it included 32 women from across England and from a variety of socio-demographic backgrounds, enabling a thematic analysis of experiences that were not linked to a single institution or area or any individual demographic group. It was a limitation that some of the initial socio-demographic diversity was lost between recruitment in pregnancy and the postnatal interviews reported here, so these participants were older, less ethnically diverse and less likely to live in disadvantaged areas than the original sample.

## 5 Conclusions

First time mothers felt uncertain and sometimes overwhelmed by the intensity of the unfamiliar challenges of becoming a mother. They described how they wanted professionals to affirm their competence as mothers by giving positive feedback, to proactively give them necessary and reliable information in a useable format, to reassure them that their baby was behaving normally, to treat them with compassion while acknowledging the magnitude of the life transition, and to enable their partner or family member to provide effective practical support. Health professionals working in postnatal care can play an important role in helping first time mothers to cope with stress of becoming a parent and to thrive, by taking every opportunity to give appropriate and personalised appraisal, informational and emotional social support alongside clinical or functional care. Training and professional leadership may help to ensure that all health professionals are able and expected to offer the positive social support already offered by some. Further research could investigate the formats of information that postnatal mothers find most useful.

## Authors’ agreement

We confirm that:

• The article is our original work.
• The article has not received prior publication and is not under consideration for publication elsewhere.
• All authors have seen and approved the manuscript being submitted.
• The authors abide by the copyright terms and conditions of Elsevier and the Australian College of Midwives.

## Funding

This research is funded by the 10.13039/501100000272National Institute for Health Research (NIHR) Policy Research Programme, conducted through the Policy Research Unit in Maternal and Neonatal Health and Care, PR-PRU-1217-21202. The views expressed are those of the authors and not necessarily those of the NIHR or the Department of Health and Social Care. The funders did not have any role in the study.

## Ethical statement

The University of Oxford Medical Sciences Inter-Divisional Research Ethics Committee (reference R52703/RE001) approved the study on 8th September 2017. Participants consented to data collection and for their experiences to be used in reports or publications with no details or other information being published that could identify them. Following the consent process the individual qualitative interview transcripts will not be made publicly available.

## Conflict of interest

None declared.

## CRediT authorship contribution statement

**Jenny McLeish:** Methodology, Investigation, Formal analysis, Writing - original draft. **Merryl Harvey:** Methodology, Investigation, Writing - review & editing. **Maggie Redshaw:** Conceptualization, Methodology, Writing - review & editing. **Fiona Alderdice:** Conceptualization, Methodology, Formal analysis, Writing - review & editing.
